# An emerging role for IQGAP1 in tight junction control

**DOI:** 10.1080/21541248.2016.1244440

**Published:** 2016-11-23

**Authors:** Barbara E. Tanos, Charles Yeaman, Enrique Rodriguez-Boulan

**Affiliations:** a Division of Cancer Therapeutics, The Institute of Cancer Research, London, UK; b Department of Anatomy and Cell Biology, The University of Iowa, Iowa City, IA, USA; c Department of Ophthalmology, Margaret Dyson Vision Research Institute, Weill Cornell Medical College, New York, NY, USA; d Department of Cell and Developmental Biology, Weill Cornell Medical College, New York, NY, USA

**Keywords:** cdc42, claudins, exocyst, IQGAP1, tight junctions

## Abstract

IQGAP1 is a scaffold protein involved in the assembly of adherens junctions. Our work has recently revealed a novel role for IQGAP1 in the regulation of tight junctions (TJ) through differential recruitment of claudins to the nascent TJ. Here, we discuss the potential mechanisms of this regulation, including IQGAP1 effects on CDC42, and IQGAP1 interactions with sorting/trafficking molecules (e.g. Exo70). Given the many roles of IQGAP1 and the large number of interacting partners, we focus our discussion of these functions in the context of junction formation, trafficking, growth factor signaling and cancer. We also propose a potential role for IQGAP1 in regulating epithelial integrity and compartmentalized signaling in epithelia.

## Introduction

Epithelial integrity and cell-to cell adhesion are established and sustained by functional protein modules that assemble adherens junctions (AJ), tight junctions (TJ) and desmosomes (see Nelson^1^). Tight junctions are vital for tissue homeostasis as they control paracellular permeability (gate function) and promote a physical separation between the apical and basolateral side of polarized cells (fence function).^2-5^ Consistent with these roles, junctions are often deregulated in a number of pathological states such as bowel inflammatory disease,^6^ polycystic kidney disease,^7^ diabetic retinopathy^8^ and macular degeneration.^9^ Epithelial structures are also often misregulated in cancer, where tissue invasion and metastasis rely on altered morphogenesis and rearrangement of junctional proteins.^10,11^ Cell-to-cell junctions need to form and dissolve at a fast pace, not only for cell migration, but to accommodate transitions between collective migration and invasion at different cancer stages.

IQGAP1 belongs to a family of scaffolding proteins that interact with signaling and structural molecules, and regulate a number of biological processes.^12-14^ It has been shown to be an effector of Ras-superfamily small GTPases, including RAS,^15^ CDC42, and RAC.^16^ In epithelial cells, IQGAP1 localizes to sites of cell–cell contact^16^ where it inhibits E-cadherin-mediated adhesion.^17,18^ Importantly, IQGAP1 is required for Ras-dependent tumorigenesis^15,19^ in an experimental model, and is highly overexpressed in a number of human tumors, suggesting that IQGAP1 can regulate aspects of oncogenic behavior. However, given its many interactors and seemingly diverse biological activities, how can IQGAP1 achieve functional specificity?

To answer this question, a more refined understanding of how IQGAP1 regulates signaling cell autonomously, and whether it can facilitate compartmentalized signaling in multi-cellular epithelia is clearly needed. Given its role in protein trafficking, we recently explored the possibility that IQGAP1 could regulate TJ formation by modulating the expression and/or localization of junctional proteins. We systematically tested this hypothesis in the model cell line MDCK,^20^ and found that IQGAP1 silencing caused a transient CDC42-dependent increase in transepithelial electrical resistance (TER) during the early stages of TJ formation.^21^ Since the strength of the IQGAP1-CDC42 interaction was found to be inversely correlated with TER during epithelia formation, we suspect that IQGAP1 has an inhibitory effect toward CDC42 during tight junction formation. Interestingly, we found that IQGAP1 knockdown (KD) resulted in enhanced claudin 4 localization to TJ and reduced claudin 2 expression and localization to TJs, suggesting this as the mechanism for the observed increase in TER. Thus, we identified IQGAP1 as an important player in the establishment of TJs, and established a mechanistic link between IQGAP1, CDC42, and the spatial regulation of claudins at the onset of epithelial polarity.

In this review, we elaborate on our finding that IQGAP1 regulates TJs, and discuss possible mechanisms of how IQGAP1 could regulate claudin trafficking to the forming junctions. Additionally, we discuss the potential role of IQGAP1 in regulating compartmentalized signaling in polarized epithelia, the potential crosstalk between growth factor receptor signaling and TJ assembly, and the implications of IQGAP1 deregulation in cancer.

## IQGAP1 and its role in cell-cell adhesion

**Figure 1.**
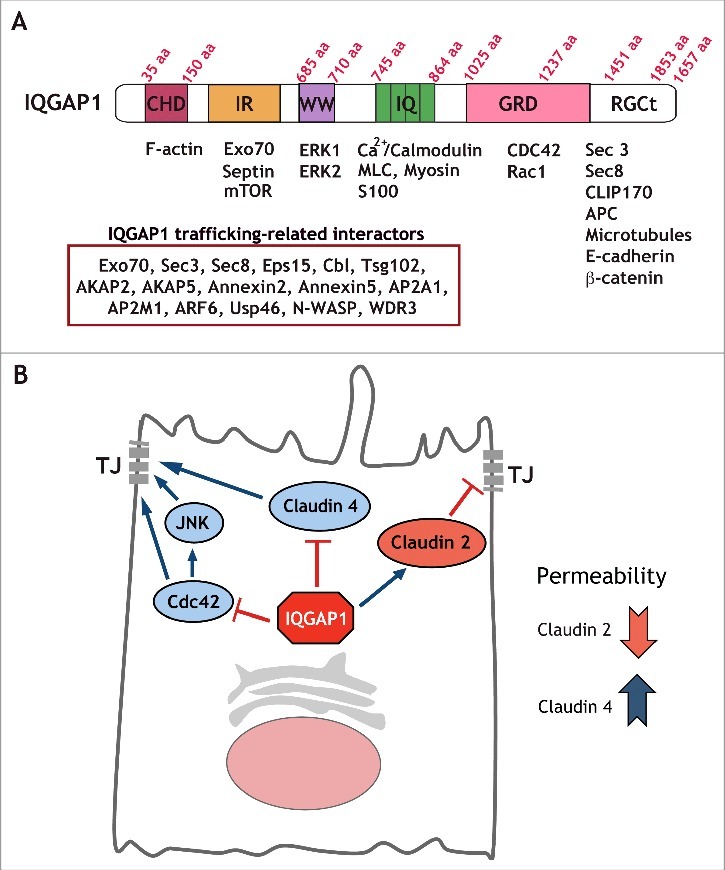
IQGAP1 interacting proteins can regulate a number of cellular functions. (A) IQGAP1 domain structure. Diagram depicting IQGAP1 domains and a number of interacting proteins. As many as 300 interactions have been documented for IQGAP1. We have listed a subset of these interactors that are relevant for cargo trafficking. (B) Model for selective regulation of TJ formation by IQGAP1. IQGAP1 promotes claudin 2 recruitment to the TJ, and blocks claudin 4 localization, thereby differentially regulating claudin localization to the forming TJ. Further, IQGAP1 also controls Cdc42 function and Cdc42/JNK activation during TJ formation.

IQGAP1 is the best characterized member of a family of scaffolding proteins that includes IQGAP1, IQGAP2, and IQGAP3 (Fig. 1). IQGAP1 regulates a number of biological processes by providing a platform for complex assembly and signal transduction (reviewed in ref. 22). Not surprisingly, its deregulation has been linked with a variety of human cancers, particularly in tumors that have lost epithelial polarity, where it is highly overexpressed.^23^ Furthermore, ectopic IQGAP1 overexpression promotes increased cell proliferation and invasion in breast epithelial cells.^23,24^

IQGAP1 localizes to sites of cell-cell contact in epithelial cells^25^ and has been shown to regulate E-cadherin-mediated cell-cell adhesion and actin reorganization.^18,25^ Specifically, IQGAP1 inhibits adherens junction formation through sequestering E-Cadherin from cell-cell contacts.^17,18^ We recently uncovered a novel role for IQGAP1 in the regulation of tight junctions.^21^ Interestingly, disruption of epithelial architecture is a common feature of IQGAP1-overexpressing tumors,^26^ consistent with the notion that epithelial polarity cues can be hijacked by cancer cells.^10^ These mispolarized epithelia are often characterized by downregulation or mislocalization of tight junction proteins such as ZO-1 and claudins.^27^ Based on these observations, we hypothesized that IQGAP1 could potentially regulate TJ formation through altering the expression or localization of TJ proteins. To examine the role of IQGAP1 in TJ formation, we measured the effects of IQGAP1 depletion on TER, an indirect readout of tight junction strength. We found that IQGAP1 knockdown promoted a significant increase in the TER peak following calcium addition during a calcium switch assay, which mimics epithelial polarization. IQGAP1 knockdown (KD) promoted an increase in junctional claudin 4 and a reduction in claudin 2 expression and TJ localization, providing a molecular explanation for the increase in TER,^21^ and illustrating a likely consequence of disrupting IQGAP1 membrane trafficking functions.^28^ Claudin 2 and claudin 4 are critical for gate function regulation. Claudin 2 increases conductivity, reducing TER through a water permeable^29^ and cation-selective pore at the TJ^30,31^ whereas claudin 4 restricts paracellular conductance reducing Na (+) permeability.^32^ Accordingly, we and others have found that in MDCK cells, depletion of claudin 2 through RNAi- or gene-targeting-mediated approaches is sufficient to promote a TER increase.^21,33^

CDC42 has been shown to be required for TJ formation.^34^ For example, knockdown of CDC42 (or its effectors PAK4 and Par6B) impairs junction formation in bronchial epithelial cells.^35^ However, increased CDC42 activation can promote E-cadherin internalization and degradation.^36^ Thus, CDC42 levels and activity have to be tightly regulated to maintain tissue homeostasis. We found that the IQGAP1-KD-mediated increase in TER could be reversed by expression of dominant negative CDC42, an interactor of IQGAP1. In addition, IQGAP1 KD promoted increased activation of JNK, a CDC42 effector. Thus, IQGAP1 may function in concert with CDC42-JNK during polarity establishment^21^ (Fig. 1). In airway epithelia, the CDC42 effector JNK has been shown to be required for TJ barrier function.^37^ Consistently, we found that IQGAP1 KD led to activation of JNK in MDCK cells, thus, providing evidence of a role for JNK in IQGAP1-regulated TJ formation.^21^

However, given the established role of IQGAP1 in destabilizing adherens junctions, how does IQGAP1 concurrently regulate tight junction formation to maintain or establish appropriate cell-to-cell contacts? In the initial steps of epithelial polarization, immature junctional patches are occupied by both E-Cadherin and ZO-1.^38^ ZO-1 subsequently moves up as TJ differentiate; the mechanisms involved in this differentiation are unknown. IQGAP1 might play a role not only by physically competing with E-Cadherin for junction occupancy, but also indirectly through regulation of E-cadherin trafficking, including E-cadherin recycling back to the plasma membrane. Since we find that IQGAP1 interacts with CDC42 throughout all cell polarization steps,^21^ we suspect that the inhibitory role of IQGAP1 in TJ formation involves sequestering CDC42 away from the junction. Interestingly, IQGAP1 has been shown form a complex with PAK6 and E-Cadherin that promotes cell dissociation in prostate cancer cells.^39^ Thus, through its scaffolding properties and binding to different junctional proteins, IQGAP1 could modulate both adherens and tight junction formation. In normal epithelia, slowing down junction formation or reducing tight junction strength might be necessary for adequate building of the junctions through temporal regulation. 

## IQGAP1 and trafficking

During epithelial polarization, additional molecules are recruited to junctional sites.

Tight junction components such as occludin and claudin are delivered to the TJ via exocytosis by the basolateral sorting machinery (reviewed in ref. 5). Interestingly, IQGAP1 has been previously shown to regulate exocytosis^40^ and may function as a regulator of protein trafficking.^28^

**Figure 2.**
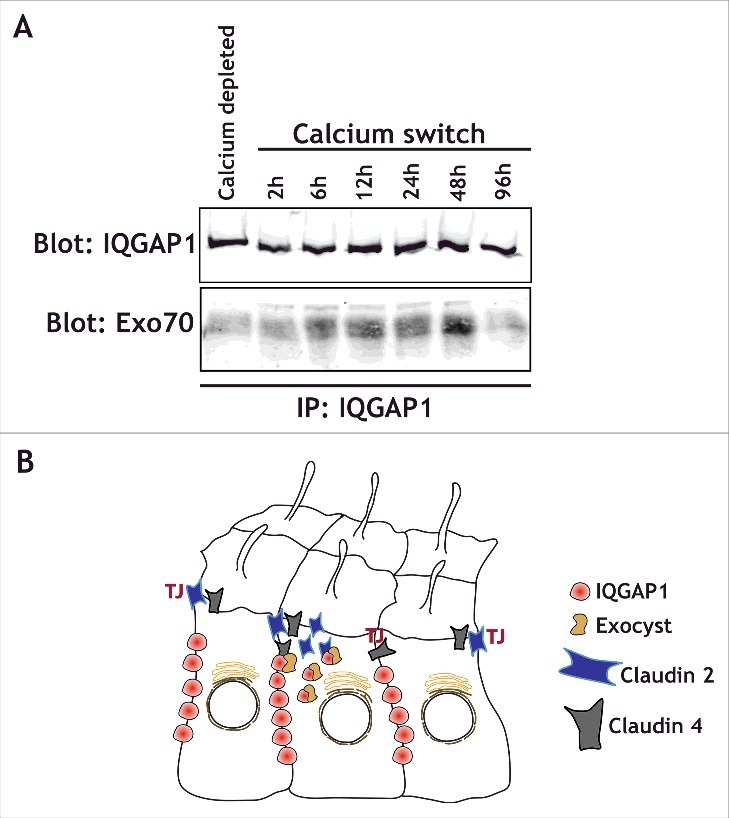
IQGAP1 interacts with the exocyst complex during TJ formation. (A) IQGAP1 immunoprecipitation in calcium depleted cells (indicated), or at different times following calcium addition in a calcium-switch experiment. Note that IQGAP1 levels are relatively unchanged throughout the experiment, but that Exo70 is mostly recruited during the establishment of the epithelia (6h to 48 hours after the calcium switch). (B) Cartoon depicting the IQGAP1/Exocyst complex interaction in the vicinity of the TJ, and its role in the differential recruitment of claudins.

**Figure 3.**
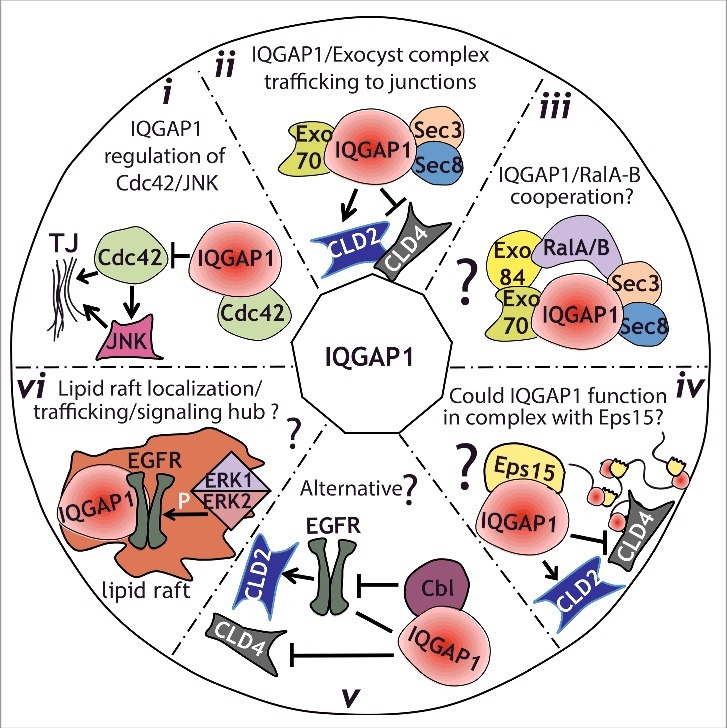
Model for IQGAP1 regulation of TJ formation. *(i)*, IQGAP1 might function by sequestering Cdc42, thereby modulating its positive influence on TJ establishment, including activation of JNK. *(ii)*, IQGAP1 interacts with the exocyst complex during TJ formation. Such interaction might regulate the sorting of specific claudins to the TJ at specific times, therefore regulating its strength. *(iii)*, IQGAP1 might function coordinately with Ral GTPases to regulate trafficking to the TJ, either through a direct interaction that has not been yet identified, or through a link provided by the exocyst complex. *(iv)*, IQGAP1 role in membrane trafficking/sorting could be facilitated by its interaction with the EH-domain-containing protein Eps15. *(v)*, an alternative hypothesis, is that IQGAP1 might sequester Cbl away from the EGFR. This, in turn, could stabilize claudin 2,^53^ resulting in an increase in Claudin 2 expression and junctional localization. *(vi)*, Since IQGAP1 has been found in lipid rafts, where it promotes EGFR phosphorylation by ERK1/2, we hypothesize that IQGAP1 could function as a signaling node and trafficking regulator in raft-like patches directing sorting/trafficking to the TJ.

We found that IQGAP1 knockdown led to a change in the distribution of claudins during epithelial polarization.^21^ Additionally, IQGAP1 is known to interact with Exo70,^40^ a component of the exocyst complex, a regulator of basolaterally directed trafficking in MDCK cells.^41^ Intriguingly, we find that in MDCK cells, IQGAP1 interacts with Exo70 during epithelial polarization shortly after calcium switch, but neither in calcium depleted, depolarized epithelia, nor 48-72 hours after a Calcium switch once epithelial polarity has been established (Fig. 2). This suggests that the interaction between IQGAP1 and Exo70 might be critical during polarization, when specific sorting is required for establishing the epithelia. Interestingly, the small GTPases RalA and RalB have been shown to regulate the exocyst complex as well as junctional assembly in epithelial cells.^42,43^ RalA and RalB specifically regulate tight junction strength and promote differential protein recruitment to the newly forming TJ.^44^ RalB knockdown phenocopied the effects of IQGAP1 knockdown, both in terms of the TER increase as well as claudin 4 and claudin 2 recruitment, while RalA knockdown had opposite effects.^44^ These observations suggest that RalA could regulate exocytosis of junctional proteins for proper localization, whereas RalB could regulate endocytosis and protein removal. Given that TJ assembly is an active and complex process, it is likely that IQGAP1 and Ral GTPases coordinately regulate protein recruitment to the forming junction. However, a specific complex between RalGTPases and IQGAP1 has not yet been demonstrated. This might be due to the time-dependent nature of this interaction. The complex might only assemble during epithelial polarization, in specific cell types, or, alternatively, it may require the exocyst to stabilize the interaction (Fig. 3).

IQGAP1 also interacts with Eps15,^45^ an EH domain containing protein which regulates trafficking in the secretory pathway.^46^ EH domain-containing proteins have been shown to regulate membrane curvature.^47^ Thus, it is tempting to speculate that IQGAP1 could modulate the secretory pathway through sorting or differential recruitment of proteins that influence membrane architecture such as Eps15 (Fig. 3).

## IQGAP1 and growth factor signaling

Where does IQGAP1 exert its effects? Our work suggests that IQGAP1 differentially regulates sorting of different proteins to the tight junction, akin to a bouncer at a club deciding who goes through and who does not. It is likely that the scaffolding properties of IQGAP1 enable these sorting/trafficking capabilities. One possibility is that lipid raft localization of IQGAP1 could generate specific patches where a specific group of trafficking vesicles is targeted to and/or sorted from. In support of this, IQGAP1 has been shown to interact with EGFR^48^ and to promote EGFR phosphorylation by ERK1/2 that has specifically been activated in lipid rafts.^49^ Interestingly, IQGAP1 has also been shown to interact with the EGFR degradation machinery,^50^ and, as mentioned above, with the trafficking regulator Eps15,^45^ also a component of recycling endosomes that modulates EGFR recycling to the plasma membrane.^51^ Therefore, one could speculate that IQGAP1 could control growth factor receptor activity by determining the endocytic fate of receptor-associated vesicles. Similarly, IQGAP1 might regulate claudin trafficking to TJ junctions through sequestering claudin 4 and allowing claudin 2 to go through. 

## IQGAP1 and cancer

In tumor cells, claudin expression and localization is modulated by the EGFR pathway, which is itself defective in claudin 2 knockout mice.^52^ These observations suggest a functional crosstalk between growth factor receptor signaling and tight junctions. Further experiments using fluorescently tagged claudins could help understand how and when IQGAP1 directs this differential distribution of claudins during TJ formation, and whether the EGFR pathway cross-modulates this process. Alternatively, IQGAP1 could function by hijacking Cbl^45^ away from EGFR, and the accompanying degradation machinery, thus preventing claudin 2 degradation and promoting accumulation at the TJ. Interestingly, our results show both a decrease in protein levels as well as TJ localization of claudin 2 in IQGAP1 deficient cells.^21^ Whether this decrease in protein expression is dependent on the lysosomal machinery, or what trafficking pathways might be involved remains to be determined. Nevertheless, some experimental evidence points to a potential model. First, it has been shown that the small GTPase rab14 controls claudin 2 levels in coordination with PKC, by promoting the removal of claudin-2 out of the lysosomal degradation pathway.^53,54^ Thus, it is possible that IQGAP1 could mediate removal of claudin 2 from the lysosomal pathway by spatially regulating the relevant rab14 complex. Alternatively (or additionally), IQGAP1 could influence the trafficking fate of claudin 2, through spatial regulation of Eps15 (Fig. 3).

Another outstanding question regarding the effects of IQGAP1 on claudin 4 is whether claudin 4 trafficking might initially be associated to the nascent junction through E-cadherin, and whether this trafficking is dependent on the exocyst complex. Additional experiments will also be needed to determine whether this regulation is directly dependent on IQGAP1, or its interactions with the exocyst complex^40^ (Fig. 2), CDC42, or both. As overexpression of claudins serves as a marker for malignant progression in some tumors,^55^ most notably breast cancer,^56^ and given the multiple links between the subcellular localization of claudins, IQGAP1, TJs, and EGFR signaling, additional studies on claudin localization in the context of claudin-overexpressing EGFR-dependent tumors is warranted.

## IQGAP1 and compartmentalized signaling

Despite the seemingly steady localization at the junction, IQGAP1 might need to relocalize fast at specific times during polarization, to be able to promote its multiple functions. One example of this is IQGAP1 removal from the site of T cell docking to cancer cells. During T cell activation, IQGAP1 moves swiftly out of the centriole docking site, along with the actin patch, allowing docking and the release of lytic granules to the cancer cell.^57^ This fast removal of IQGAP1 (and actin) immediately generates an asymmetric compartment with fast vesicle movement similar to a cilia transition zone.^57,58^ Tight junctions also define an asymmetric border, apical vs basolateral, where not only protein expression is different, but protein secretion, receptor activation and trafficking. Thus, TJs enable compartmentalized signals in polarized epithelia, where the apical and basolateral plasma membrane domains are physically separated, and molecularly and functionally distinct. In both, tight junctions and the immunological synapse, IQGAP1 may function as a molecular filter for asymmetric signal transduction. In support of this view, IQGAP1 has been shown to interact with Septin 2,^40^ which functions as a diffusion barrier for protein trafficking to the ciliary transition zone.^59^

Furthermore, by regulating the speed at which TJs form or immune cells dock, or through temporal and spatial modulation of vesicular trafficking and growth factor receptor activation, IQGAP1 could function as a quality control (QC) mechanism. This QC mechanism likely requires tightly regulated IQGAP1 levels, as IQGAP1 overexpression (e.g. during cancer) leads to decreased junctional strength and disrupted cell architecture. Supporting this point, we have found that IQGAP1 KD cells showed increased columnar appearance and a significant increase in cell height, probably as a result of increased junctional strength.^21^

## Final thoughts

Our recent work provided first-time evidence of an inhibitory role for IQGAP1 in TJ formation during epithelial polarization.^21^

Our data support a model of TJ formation that involves IQGAP1-sensitive differential recruitment of claudin 2 and claudin 4 to the TJ site, and IQGAP1-sensitive activation of CDC42/JNK that likely facilitates junction maturation (Fig. 1). Considering the clinical utility of claudin expression as a prognostic marker in variety of human cancers^60-62^ and the link between TJ disruption and metastatic disease,^63^ our work suggests that IQGAP1 could be a potential therapeutic target in advanced cancers. Of note, the remaining IQGAP family members, namely IQGAP2 and IQGAP3, have also been implicated in cancer^24,64^ and could potentially regulate junction formation, either alone or together with IQGAP1 or its binding partners.

The growing number of IQGAP1-interacting proteins and the variety of regions within this large molecule to which they bind suggest that perhaps distinct IQGAP1 domains may be functional at different times and/or cellular locations resulting in different trafficking outcomes. This could account for a number of promiscuous IQGAP1 functions that depend on cellular context (e.g., tumor type) and ultimately result in asymmetric signal transduction, and compartmentalized signaling at the cellular and tissue level.

## Abbreviations

AJ: adherens junctions
EGFR: Epidermal growth factor receptor
KD: knockdown
MDCK: Madin-Darby canine kidney
TER: transepithelial electrical resistance
TJ: Tight junctions
